# Effect of Undersized Drilling and Sequential Osseodensification on Primary Implant Stability in Simulated Bone Types III and IV: An In Vitro Study

**DOI:** 10.1155/ijod/8114317

**Published:** 2026-08-19

**Authors:** Silvio Yoshiaki Okagawa, Alex Sandro de Souza, Paulo Sérgio Perri de Carvalho, Antonio Scarano, Bruno Freitas Mello, Sergio Rexhep Tari, Gustavo Coura, Gustavo Vicentis Oliveira Fernandes, Sergio Alexandre Gehrke

**Affiliations:** ^1^ Department of Implant Dentistry, SLMANDIC - Sao Leopoldo Mandic, Campinas 13045–755, Brazil, slmandic.edu.br; ^2^ Department of Innovative Technologies in Medicine & Dentistry, University of Chieti-Pescara, Chieti 66013, Italy, unich.it; ^3^ Department of Pharmaceutical Science, School of Health Sciences, Vale do Itajai University (UNIVALI), Itajai 88302–901, Brazil, univali.br; ^4^ Department of Implantology, Be Dental School, Vale do Itajai University (UNIVALI), São Jose 88102–700, Brazil, univali.br; ^5^ Department of Implantology, Bioface/Catolica de Murcia University, Montevideo 11100, Uruguay; ^6^ Missouri School of Dentistry and Oral Health, A.T. Still University, St. Louis 63104, Missouri, USA, atsu.edu

**Keywords:** implant stability, low-density bone, osseodensification, polyurethane model, torque, undersized drilling

## Abstract

**Purpose:**

Primary implant stability is a key factor for successful osseointegration and may be influenced by osteotomy preparation techniques, particularly in low‐density bone. Thus, this study compared undersized drilling (UD), UD combined with osseodensification, and sequential osseodensification protocols with varying degrees of undersizing in simulated bone types III and IV with respect to implant stability. Additionally, the relationships between insertion torque (IT), removal torque (RT), and implant stability quotient (ISQ) were analyzed.

**Materials and Methods:**

Eighty implants (4.0 × 11 mm) were placed in polyurethane blocks simulating type III and IV bone with a cortical layer. Four osteotomy protocols were tested: UD, UD plus osseodensification (OD1), and sequential osseodensification with −0.5 mm (OD2) and −0.7 mm (OD3) undersizing. Primary stability was assessed using IT, RT, and ISQ measurements. Data were analyzed using two‐way ANOVA, Pearson correlation, and multiple regression analysis (*α* = 0.05).

**Results:**

OD2 and OD3 produced higher IT values in both bone types compared with UD and OD1. In type III bone, OD3 showed the highest RT values, while in type IV bone, OD2 and OD3 were significantly higher than UD and OD1. ISQ values were higher for osseodensification protocols in type III bone, whereas no significant differences were observed in type IV bone. A strong correlation was found between IT and RT (*r* = 0.746), whereas ISQ showed weaker associations with torque.

**Conclusion:**

The use of sequential osseodensification protocols yielded superior mechanical stability parameters, specifically in IT and RT, in synthetic (polyurethane) bone models. However, improvements in ISQ were predominantly noted in the denser type III model. Importantly, because the tested protocols involved different drill designs and underpreparation dimensions, these enhancements reflect the success of the overall surgical strategy rather than the isolated effect of osseous densification.

## 1. Introduction

Osseointegration is the direct structural and functional connection between the living bone and a load‐bearing implant, providing immediate mechanical stability and supporting long‐term success [1]. Successful osseointegration depends on bone quality, surgical technique, and implant material. Titanium, widely used in dental implants, forms a stable oxide layer that prevents ionic leakage and protects surrounding tissues, allowing direct bone contact and promoting mechanical engagement at the implant‐bone interface [2].

Primary stability is a key determinant of early osseointegration, particularly in low‐density bone. It is influenced primarily by bone quality, osteotomy technique, and osteotomy sizing relative to the implant. According to Lekholm and Zarb [3], type III bone presents a thin cortical layer over low‐density trabecular bone, whereas type IV bone features thin cortical bone surrounding sparse trabeculae, commonly found in the posterior maxilla. Achieving adequate primary stability in these conditions remains a clinical challenge.

Conventional drilling protocols in low‐density bone often employ underpreparation, creating an osteotomy slightly smaller than the implant (~10%) to improve mechanical engagement [4]. More recently, the osseodensification technique, introduced by Huwais and Meyer [5], uses specially designed drills to compact and preserve bone rather than remove it, exploiting the viscoelastic and plastic properties of trabecular bone. This compaction increases bone density within the osteotomy and may enhance primary implant stability.

Primary implant stability is commonly evaluated using insertion torque (IT), removal torque (RT), and resonance frequency analysis (RFA), which are combined to yield the implant stability quotient (ISQ). Although these parameters are frequently reported in implant research, they represent distinct biomechanical aspects of the implant‐bone interface [6, 7]. IT reflects the mechanical resistance encountered during implant placement and is often associated with bone density and implant–site preparation [8]. RT, on the other hand, represents the resistance required to disrupt the mechanical interlocking between the implant surface and the surrounding substrate [9, 10]. ISQ values obtained through RFA, commonly measured using systems such as Osstell, reflect the stiffness of the implant–bone complex rather than the rotational resistance at the interface [11].

Despite their widespread use, the relationship between IT, RT, and ISQ remains controversial. Several studies have reported that higher IT does not necessarily translate into higher ISQ values, particularly in low‐density bone or when different osteotomy techniques are employed [12, 13]. This suggests that these parameters may capture different biomechanical characteristics of primary implant stability. Evaluating the correlations among IT, RT, and ISQ may provide additional insights into the biomechanical behavior of the implant‐site interface beyond simple group comparisons. Investigating whether IT or RT can predict ISQ values may help clarify which mechanical factors most strongly influence primary stability. Thus, while IT measures the rotational friction encountered during osteotomy preparation and RT quantifies the force required to shear the established implant‐substrate interface, RFA evaluates the lateral micromobility and stiffness of the system, yielding an ISQ. Clinically, IT ≥ 30 Ncm and ISQ ≥ 60 are frequently cited as acceptable thresholds for immediate loading protocols. Comparing these three unique metrics provides a highly comprehensive biomechanical profile that a single parameter cannot capture.

The specialized macrogeometry of osseodensification drills, characterized by multiple flutes and a negative rake angle, facilitates mechanical compaction of the substrate walls rather than their excision. To systematically evaluate this, the current study assessed four distinct osteotomy strategies (undersized drilling [UD], UD + osseodensification, and sequential osseodensification drilling with −0.5 and 0.7 mm of undersizing) on primary implant stability across varying bone density analogs (type III and type IV bone). Specifically, the study compared IT, RT, and ISQ across techniques and bone types and explored their interrelationships using correlation and regression analyses to elucidate the biomechanical determinants of stability. The null hypothesis was that there would be no significant differences in IT, RT, or ISQ across the distinct osteotomy protocols or simulated bone densities.

## 2. Materials and Methods

This study followed the CRIS guidelines for in vitro studies.

### 2.1. Experimental Model

Synthetic polyurethane blocks (*n* = 8, dimensions: 95 mm × 45 mm× 30 mm, Nacional Ossos, Jaú, Brazil) were selected to replicate human bone morphology. These synthetic analogs comply with the American Society for Testing and Materials (ASTM) F1839‐08 standard, ensuring high consistency and eliminating the anatomical variability inherent in cadaveric or animal models [14]. The models were stratified into two categories matching the Lekholm and Zarb classification [3]: type III simulated bone (four blocks) (pink; 20 PCF; 0.32 g/cm^3^) and type IV simulated bone (orange; 15 PCF; 0.24 g/cm^3^). All blocks included a 2‐mm high‐density superficial layer (brown; 40 PCF; 0.48 g/cm^3^) to simulate cortical bone.

### 2.2. Sample Size and Experimental Groups

An a priori power analysis was conducted using G
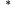
Power software (v. 3.1, Heinrich‐Heine‐Universität Düsseldorf, Germany), informed by large effect sizes reported in previous ex vivo biomechanical investigations evaluating implant stability in polyurethane models [7, 10]. Assuming an effect size (f) of 0.40, the calculation determined that a sample size of 10 osteotomies per subgroup would provide greater than 80% power to detect significant differences at an alpha level of 0.05. Each subgroup comprised 10 osteotomies/implants, for a total of 40 osteotomies per bone‐density group. Therefore, a total of 80 external hex implants (Maestro, Implacil/Osstem, São Paulo, Brazil), measuring 4 mm in diameter and 11 mm in length, were used. The implants were used solely as a standardized component to evaluate the effects of different osteotomy techniques and were not compared with respect to implant design or performance.

The groups were distinctly subdivided: (1) UD group: conventional UD (Implacil kit; Implacil/Osstem, São Paulo, Brazil); (2) OD1 group: UD followed by localized densification (Implacil kit; Implacil/Osstem, São Paulo, Brazil); (3) OD2 group: sequential osseodensification with −0.5 mm undersizing (Maximus, Contagem, Brazil); and (4) OD3 group: sequential osseodensification with −0.7 mm undersizing (Maximus, Contagem, Brazil).

### 2.3. Osteotomy Preparation

Drilling procedures were performed using a BLM 600 Driller motor (Driller, São Paulo, Brazil) coupled to a 20:1 reduction contra‐angle handpiece, operating at 900 rpm and 20 Ncm torque, with continuous irrigation of distilled water at room temperature. All procedures were performed by a single operator to minimize procedural variability. A minimum distance of 10 mm was maintained between osteotomies to prevent mechanical interference.

In the UD group, osteotomies were prepared using a 2.0 mm lance drill followed by a 3.5 mm tapered drill, both to a depth of 11.5 mm. In the Implacil osseodensification protocol (OD1 group), the same drilling sequence was used as in the UD group (2.0 mm lance drill followed by a 3.5 mm tapered drill), followed by clockwise osseodensification with a −0.4 mm.

**Figure 1:**
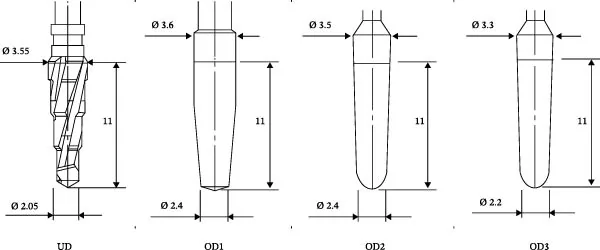
Schematic representation of the final drill used for osteotomy preparation in each experimental group. Instruments corresponding to the conventional undersized drilling (UD) protocol, the Implacil osseodensification protocol (OD1), and the progressive osseodensification protocols (OD2 and OD3) are shown with their respective diameters (Ø) and final depths. All dimensions are expressed in millimeters.

**Figure 2:**
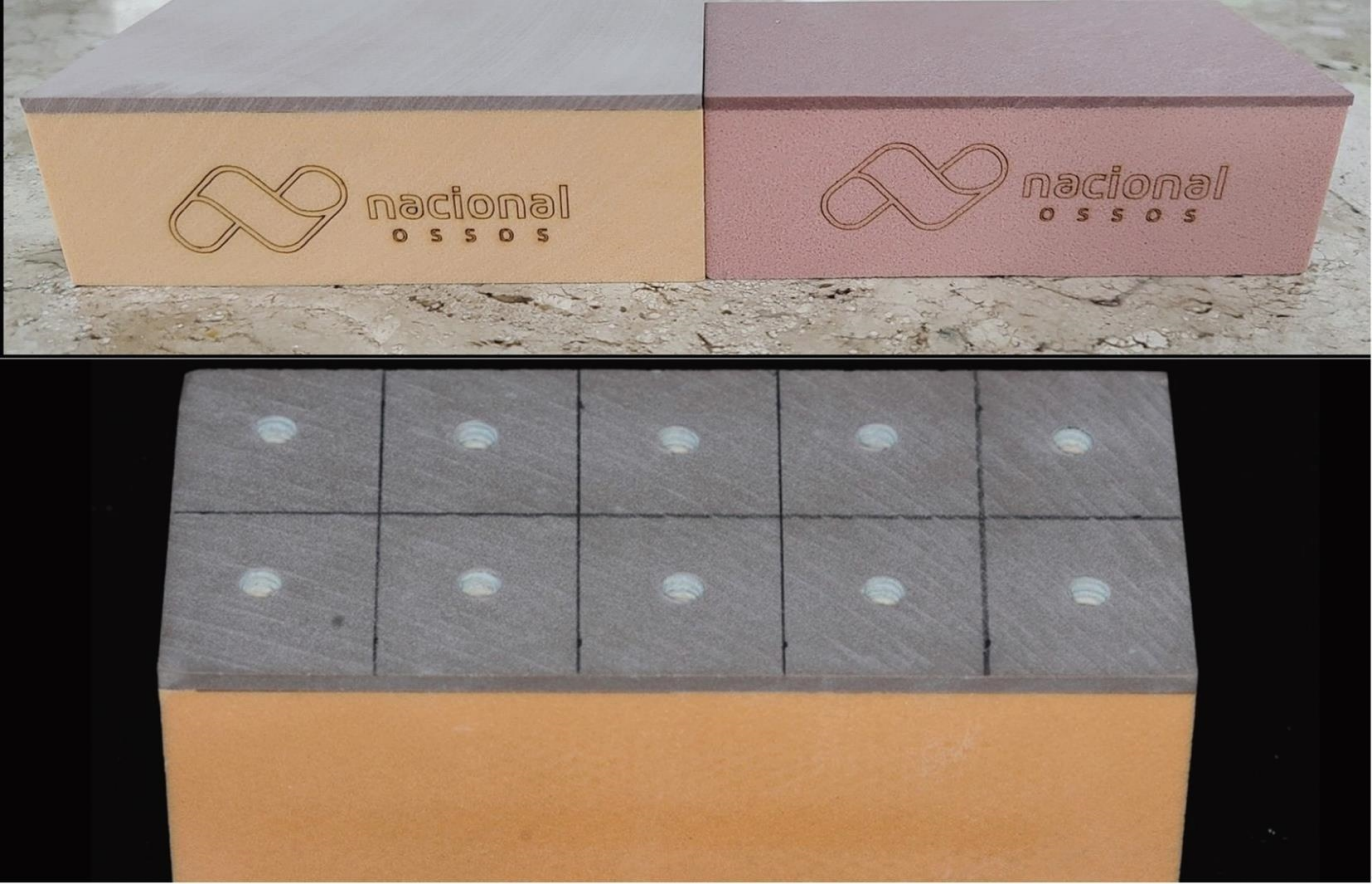
The synthetic polyurethane blocks before and immediately after preparation.

For the OD2 and OD3 groups, a 1.0 mm drill was used. This was followed by a progressive osseodensification drill sequence (1.6, 1.8, 2.0, 2.3, 2.5, 2.8, 3.0, and 3.3 mm) for the OD3 group (intended −0.7 mm undersizing) and the identical sequence plus an additional 3.5 mm osseodensification drill for the OD2 group (intended −0.5 mm undersizing). The entire osseodensification sequence was performed clockwise. Figure 1 schematically illustrates the design and dimensions of the final instrument used for osteotomy preparation in each group. The synthetic polyurethane blocks before and immediately after preparation are demonstrated in Figure 2.

### 2.4. Implant Stability Measurements

Implants were inserted using a digital torque wrench (Homis, São Paulo, Brazil) coupled to a 1.2 mm internal hex driver, and the final IT values were recorded. Primary stability was assessed using RFA with the Osstell ISQ system (Osstell AB, Gothenburg, Sweden). A SmartPeg transducer was attached to each implant, and four measurements were obtained in the buccal, lingual, mesial, and distal directions. RT was then measured using the Homis digital torque wrench by applying counterclockwise rotation until the implant began to rotate within the polyurethane block.

### 2.5. Statistical Analysis

Statistical analyses were performed using the IBM SPSS Statistics software (version 16; IBM Corp., Armonk, NY, USA). Descriptive statistics were expressed as mean ± standard deviation (SD). Prior to parametric testing, linear regression assumptions were verified using the Shapiro–Wilk test for residual normality, Levene’s test for homoscedasticity, and the variance inflation factor (VIF) thresholds to rule out multicollinearity. The effects of osteotomy technique and bone type on IT, RT, and ISQ were evaluated using two‐way analysis of variance (ANOVA), followed by Tukey post hoc tests when significant interactions were detected. Pearson correlation analysis was performed to examine the relationships between IT, RT, and ISQ values. Multiple linear regression analysis was conducted to evaluate the influence of IT and RT on ISQ values, and the coefficient of determination (*R* ^2^) was calculated. The coefficient of variation (CV) was computed to assess measurement consistency. A significance level of *p* < 0.05 was adopted for all statistical tests.

## 3. Results

**Figure 3:**
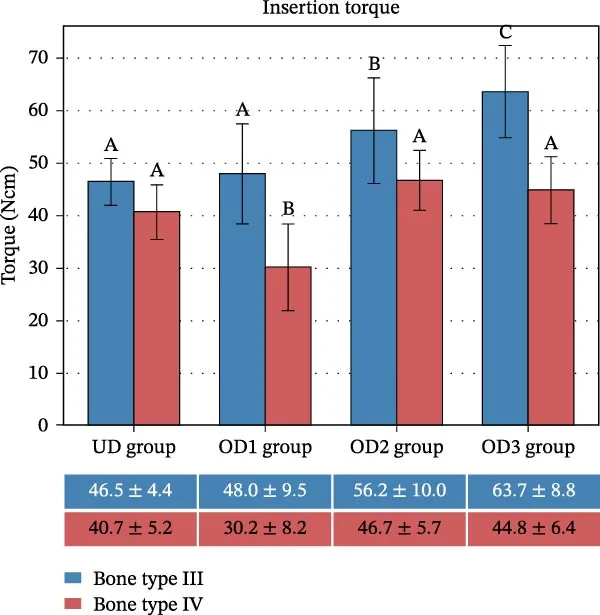
Insertion torque (IT, Ncm) according to osteotomy technique (UD, OD1, OD2, OD3) and simulated bone type (type III and type IV). Bars represent mean ± SD. Different uppercase letters indicate statistically significant differences among osteotomy techniques within the same bone type (*p* < 0.05). The table below the graph shows the mean ± SD values for each group.

Two‐way ANOVA revealed a significant interaction between osteotomy technique and bone type for IT (*F* = 6.345, *p* < 0.001). In type III bone, the OD3 group showed the highest IT values, significantly higher than those of the OD1 group (*p* < 0.0001) but not significantly different from those of the OD2 group (*p* = 0.0557). The OD2 group also produced higher IT than the UD group (*p* < 0.0001). In type IV bone, both the OD2 and OD3 groups showed significantly higher IT than the OD1 group (*p* < 0.0001 and *p* = 0.0001, respectively), whereas no significant difference was observed between OD2 and OD3. Figure 3 illustrates the distribution of IT according to osteotomy technique and bone type. Overall, the OD2 and OD3 groups generated higher IT values, particularly in lower‐density bone.

### 3.1. RT

**Figure 4:**
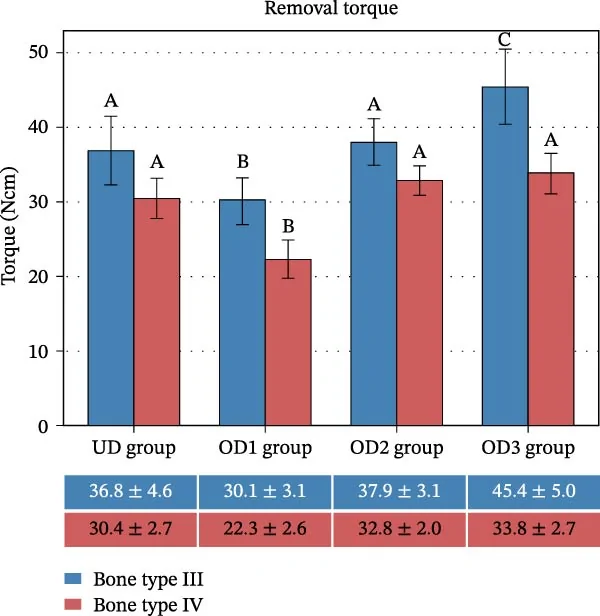
Removal torque (RT, Ncm) according to osteotomy technique and bone type. Bars represent mean values, and error bars indicate standard deviation (SD). Different letters above the bars indicate statistically significant differences among osteotomy techniques within the same bone type (*p* < 0.05). The table below the graph shows the mean ± SD values for each group.

Two‐way ANOVA also indicated a significant interaction between groups and bone type for RT (*F* = 3.822, *p* < 0.0001). In type III bone, the OD3 group yielded the highest RT, significantly exceeding all other techniques (*p* < 0.0001), while the OD1 group showed the lowest RT (*p* < 0.0001). In type IV bone, RT did not differ between the OD2 and OD3 groups (*p* = 0.895), but both were significantly higher than in the UD and OD1 groups (*p* < 0.0001). Figure 4 depicts RT distributions across techniques and bone types. These results indicate that the preparation of the OD2 and OD3 groups enhances mechanical resistance at the implant‐bone interface.

### 3.2. ISQ

A significant interaction between osteotomy technique and bone type was observed for ISQ values (*F* = 18.94, *p* < 0.0001).

In type III bone, the UD group showed the lowest ISQ values and differed significantly from the OD1 group (*p* = 0.0248) and the OD2 group (*p* = 0.0347).

**Figure 5:**
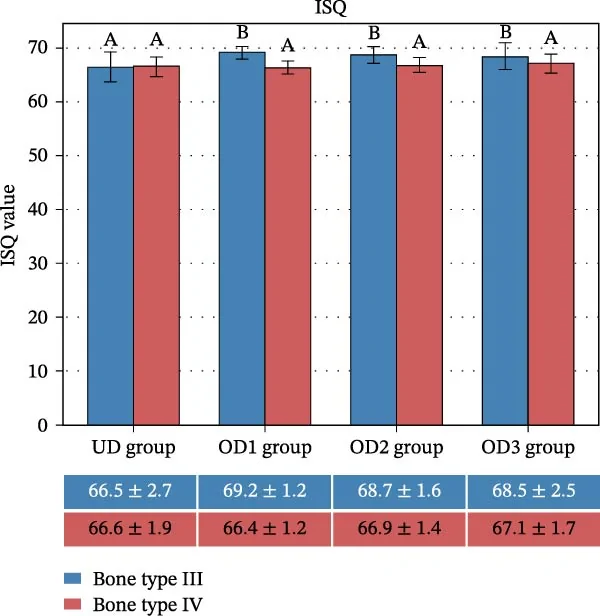
Implant stability quotient (ISQ) according to osteotomy technique and bone type. ISQ values represent the mean of four measurements (buccal, lingual, mesial, and distal) obtained using resonance frequency analysis with the Osstell system. Bars represent mean values, and error bars indicate standard deviation (SD). Different letters above the bars indicate statistically significant differences among osteotomy techniques within the same bone type (*p* < 0.05). The table below the graph shows the mean ± SD values for each group.

In type IV bone, ISQ values did not differ significantly among groups. Figure 5 shows ISQ by osteotomy technique and bone type. Overall, OD groups (osseodensification) improved ISQ in type III bone, whereas no clear effect was observed in type IV.

### 3.3. Correlation and Regression Analysis

**Figure 6:**
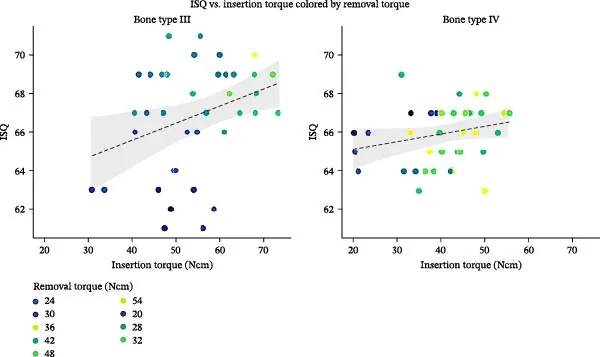
Scatter plots illustrate the relationship between insertion torque (IT) and implant stability quotient (ISQ) for bone types III and IV. Point colors represent removal torque (RT, Ncm) values. Gray dashed lines indicate linear regression trends. This figure illustrates the combined biomechanical relationship between IT, RT, and implant stability.

Pearson correlation analysis revealed a strong positive correlation between IT and RT (*r* = 0.746, *p* < 0.001), a moderate correlation between IT and ISQ (*r* = 0.345, *p* = 0.002), and a weak, nonsignificant correlation between RT and ISQ (*r* = 0.185, *p* = 0.103). Figure 6 illustrates the relationship between IT and ISQ for bone types III and IV, with point colors representing RT values. Gray dashed lines indicate linear regression trends, highlighting that ISQ is more strongly influenced by RT than IT, particularly in bone type IV (Figure S1).

Multiple linear regression of ISQ on IT and RT yielded the equation ISQ = 61.213–0.006 × IT + 0.161 × RT. The model exhibited modest explanatory power, accounting for a minor portion of the variance (*R* ^2^ = 0.23). Given the weak and non‐significant correlation between RT and ISQ (*r* = 0.185, *p* = 0.103), the data indicate that neither IT nor RT can be considered a strong predictor of ISQ within the limitations of the present study.

## 4. Discussion

This study evaluated the effects of different osteotomy techniques on primary implant stability in polyurethane blocks simulating bone types III and IV. The present data indicate that protocols incorporating sequential densification (OD2 and OD3) generally maximized rotational friction metrics (IT and RT). Recent studies heavily align with our mechanical findings, confirming that osseodensification safely augments initial stability and supports ridge expansion [15–17].

### 4.1. IT and Mechanical Effects of Osseodensification

IT progressively increased from conventional drilling to Implacil osseodensification and further with the Maximus undersized protocols, particularly in type III bone. Type IV bone showed a similar trend, though significant differences were mostly observed between the OD2 and OD3 groups. These results align with the principle that conventional drilling removes bone, whereas osseodensification compacts it, increasing mechanical resistance during implant placement.

Preclinical and ex vivo studies support these findings, demonstrating that osseodensification enhances IT compared with subtractive techniques, especially in low‐density bone [7, 18]. According to previous literature, theoretical biological mechanisms such as the compaction of bone walls and the preservation of autologous bone chips created by osseodensification can increase implant‐bone contact and mechanical engagement [5]. While our polyurethane analog model cannot directly demonstrate these biological phenomena, these proposed mechanisms from the literature may theoretically explain the higher mechanical resistance and IT values observed in clinical and in vivo settings.

### 4.2. RT and Predictors of Stability

RT followed a pattern similar to IT, with the OD3 group producing the highest values in type III bone and significantly higher RT than the UD and OD1 groups. In type IV bone, both the OD2 and OD3 groups showed higher RT than the UD and OD1 groups. The moderate‐to‐strong correlation between IT and RT (*r* = 0.746, *p* < 0.001) indicates that both parameters reflect the mechanical interlocking of the implant with the surrounding bone, consistent with biomechanical principles described in the literature [7, 8].

### 4.3. ISQ and Biomechanical Interpretation

ISQ values were generally higher in type III bone, with osseodensification showing modest improvements compared with conventional drilling. In type IV bone, no significant differences were observed among groups. The moderate correlation between IT and ISQ (*r* = 0.345, *p* = 0.002) and the weak correlation between RT and ISQ (*r* = 0.185, *p* = 0.103) highlight that RFA captures aspects of stiffness and implant–bone interface rigidity that are not fully explained by IT or RT alone [19–21].

Multiple linear regression analysis yielded a model with modest explanatory power (*R* ^2^ = 0.23). Given the weak and non‐significant correlation between RT and ISQ (*r* = 0.185, *p* = 0.103), it is evident that neither IT nor RT serves as a strong predictor of ISQ within the confines of this experimental model. These observations are consistent with previous clinical and preclinical studies showing that IT and ISQ correlate positively but may not increase in proportion across all bone densities or drilling protocols [22–24].

### 4.4. Influence of Bone Density

Bone density significantly modulated the effects of osteotomy techniques. Type III bone exhibited greater differences in IT and RT across techniques, whereas type IV bone showed smaller variations, suggesting that the mechanical benefits of osseodensification are more pronounced in low‐ to moderate‐density bone. Clinical studies support this finding, reporting that denser bone generally yields higher torque and ISQ values, whereas low‐density bone benefits more from compaction strategies [25–27].

### 4.5. Clinical and Research Implications

From a clinical perspective, the favorable mechanical stability observed with sequential osseodensification protocols may be particularly relevant in anatomically challenging situations characterized by limited bone volume and reduced bone density. Clinical evidence has demonstrated that osseodensification can facilitate simultaneous implant placement and ridge expansion in narrow anterior maxillary ridges while maintaining peri‐implant bone levels and enhancing implant stability [28]. Although the present in vitro findings cannot be directly extrapolated to clinical outcomes, they support the concept that osteotomy preparation strategies incorporating osseodensification may contribute to predictable implant stabilization and may offer advantages in cases where primary stability is critical for successful treatment and esthetic rehabilitation.

These findings motivate future research to translate static ex vivo data into dynamic biological models. Future investigations should prioritize in vivo histological studies to measure actual bone‐to‐implant contact (BIC) and evaluate the cellular remodeling response surrounding densified autogenous bone chips. Isolating drill geometry from underpreparation dimensions in future randomized trials will be essential to definitively isolate the true biological advantages of osseodensification.

### 4.6. Limitations of the Study and Confounding Variables

It is critical to interpret these findings within the context of the experimental design. The tested subgroups varied not only in the application of osseodensification but also in the final osteotomy diameter, the degree of undersizing, and the specific drill manufacturer used (Implacil vs. Maximus). Consequently, the superior mechanical performance of the OD2 and OD3 groups represents the success of an entire surgical strategy rather than the isolated mechanistic effect of densification alone. Furthermore, polyurethane blocks test only mechanical displacement; they lack the trabecular architecture, viscoelasticity, and remodeling potential of vital bone.

## 5. Conclusions

Within the limitations of this study, osteotomy preparation strategies involving sequential osseodensification produced higher IT and RT values than conventional UD protocols. However, because the evaluated groups differed not only in drilling technique but also in osteotomy geometry and the degree of underpreparation, the observed differences cannot be attributed solely to the osseodensification process. Rather, the results reflect the combined effect of the overall surgical preparation strategy. ISQ values demonstrated smaller and less consistent differences among groups, although they provided complementary information when interpreted alongside IT and RT measurements. These findings suggest that sequential osseodensification protocols may enhance the mechanical stability of implant placement in low‐density bone conditions, but further studies controlling for osteotomy dimensions are required to isolate the specific contribution of osseous densification.

## Author Contributions

**Silvio Yoshiaki Okagawa and Alex Sandro de Souza**: conceptualization, methodology, validation, formal analysis, investigation, resources, data curation, writing – original draft, writing – review and editing, visualization. **Paulo Sérgio Perri de Carvalho and Antonio Scarano**: conceptualization, methodology, software, validation, formal analysis, investigation, data curation, writing – original draft, writing – review and editing, visualization. **Bruno Freitas Mello**: conceptualization, methodology, validation, formal analysis, investigation, resources, data curation, writing – original draft, writing – review and editing, visualization, project administration. **Sergio Rexhep Tari**: conceptualization, methodology, validation, formal analysis, investigation, resources, data curation, writing – original draft, writing – review and editing, visualization, supervision. **Gustavo Coura**: conceptualization, methodology, validation, formal analysis, investigation, data curation, writing – original draft, writing – review and editing, visualization. **Sergio Alexandre Gehrke**: conceptualization, methodology, software, validation, formal analysis, investigation, resources, data curation, writing – original draft, writing – review and editing, visualization, supervision, project administration. **Gustavo Vicentis Oliveira Fernandes**: conceptualization, methodology, software, validation, investigation, data curation, writing – original draft, writing – review and editing, visualization.

## Funding

This study did not receive any specific funding.

## Disclosure

All authors have read and approved the final version of the manuscript. Sergio Alexandre Gehrke and Gustavo Vicentis Oliveira Fernandes had full access to all of the data in this study and take complete responsibility for the integrity of the data and the accuracy of the data analysis. All authors have read and agreed to the published version of the manuscript.

## Ethics Statement

The authors have nothing to report.

## Consent

The authors have nothing to report.

## Conflicts of Interest

The authors declare no conflicts of interest.

## Supporting Information

Additional supporting information can be found online in the Supporting Information section.

## Supporting information


**Supporting Information** Figure S1: Coefficient of variation (CV, %) for insertion torque (IT), removal torque (RT), and implant stability quotient (ISQ) according to osteotomy technique and bone type. The CV was calculated as (standard deviation ÷ mean) × 100. This analysis illustrates measurement consistency across techniques, showing higher variability in osseodensification protocols in bone type IV, whereas ISQ measurements remained consistently low across all groups. The CV confirmed measurement consistency: ISQ CV: 1.6–3.2% (low variability across all groups); IT and RT CV: 6–27%, with the highest variability observed in the OD1 group for type IV bone. The figure depicts the CVs for IT, RT, and ISQ across groups and bone types, highlighting consistent ISQ measurements and greater variability in torque measures for some groups.

## Data Availability

The authors confirm that the data supporting the findings of this study are available within the article and/or its Supporting Information.
